# Evaluation of the NLRP3 Inflammasome Activating Effects of a Large Panel of TiO_2_ Nanomaterials in Macrophages

**DOI:** 10.3390/nano10091876

**Published:** 2020-09-19

**Authors:** Julia Kolling, Jonas Tigges, Bryan Hellack, Catrin Albrecht, Roel P. F. Schins

**Affiliations:** 1 IUF—Leibniz Research Institute for Environmental Medicine, Auf’m Hennekamp 50, 40225 DE Düsseldorf, Germany; julia.kolling@gmx.de (J.K.); jonas.tigges@gmx.de (J.T.); catrin.albrecht@sachsen-anhalt.de (C.A.); 2 Bundeswehr Institute of Pharmacology and Toxicology, Neuherbergstraße 11, 80937 DE Munich, Germany; 3 Institute of Energy and Environmental Technology e.V. (IUTA), Bliersheimer Str. 60, 47229 Duisburg, Germany; bryan.hellack@uba.de; 4 UBA—German Environment Agency, Paul-Ehrlich-Str. 29, 63225 Langen, Germany; 5 State Office for Consumer Protection Saxony-Anhalt, 39576 Stendal, Germany

**Keywords:** nanomaterials, titanium dioxide, NALP3, interleukin-1beta, NR8383, bone marrow derived macrophages

## Abstract

TiO_2_ nanomaterials are among the most commonly produced and used engineered nanomaterials (NMs) in the world. There is controversy regarding their ability to induce inflammation-mediated lung injuries following inhalation exposure. Activation of the NACHT, LRR and PYD domains-containing protein 3 (NALP3) inflammasome and subsequent release of the cytokine interleukin (IL)-1β in pulmonary macrophages has been postulated as an essential pathway for the inflammatory and associated tissue-remodeling effects of toxic particles. Our study aim was to determine and rank the IL-1β activating properties of TiO_2_ NMs by comparing a large panel of different samples against each other as well as against fine TiO_2_, synthetic amorphous silica and crystalline silica (DQ12 quartz). Effects were evaluated in primary bone marrow derived macrophages (BMDMs) from NALP3-deficient and proficient mice as well as in the rat alveolar macrophage cell line NR8383. Our results show that specific TiO_2_ NMs can activate the inflammasome in macrophages albeit with a markedly lower potency than amorphous SiO_2_ and quartz. The heterogeneity in IL-1β release observed in our study among 19 different TiO_2_ NMs underscores the relevance of case-by-case evaluation of nanomaterials of similar chemical composition. Our findings also further promote the NR8383 cell line as a promising in vitro tool for the assessment of the inflammatory and inflammasome activating properties of NMs.

## 1. Introduction

Due to their unique properties, engineered nanomaterials (NMs) have been used since many decades in several different applications. With their increasing production and potential exposure, there is also rising concern about possible harmful properties of these compounds regarding human health. This is also the case for titanium dioxide (TiO_2_) NMs; they are among the most produced nanomaterials worldwide and are applied in a large variety of sectors including agriculture, energy, the food and cosmetic industries as well as in chemical and biomedical research [1,2]_._

Inhalation of crystalline silica is well known to trigger lung inflammation. And with high and persistent exposures to these mineral dust particles, the resulting sustained inflammation is implicated in the development of deliberating lung diseases including silicosis and cancer [3]. The inflammatory effects of crystalline silica particles are driven by their surface chemistry and ability to generate reactive oxygen species (ROS) and associated oxidative stress [4,5]. Compared to crystalline silica, TiO_2_ particles are traditionally thought to have a relatively low toxicity. Yet, there is concern for possible adverse health effects of TiO_2_ NMs, which has been linked to ROS generation and inflammatory processes upon inhalation as well [6,7,8]. It has been shown that particle size and surface are important characteristics of TiO_2_ with particles in a nano-scale (20 nm) being much more cytotoxic than fine TiO_2_ (250 nm), driven by their increased specific surface area dose [9,10]. Other acute inhalation studies detected microvascular dysfunctions and peripheral vascular effects with nano TiO_2_ being six to seven times more reactive than fine TiO_2_ [11]. Normalization of the dose on particle surface area basis resulted in an equal potency for fine- and nano-TiO_2_. 

With the identification of the “NACHT, LRR and PYD domains-containing protein 3” (NALP3) inflammasome as an essential component in crystalline silica induced lung inflammation and fibrosis [12,13,14] it has also been proposed that this pathway dominates the inflammatory properties of inhaled nanomaterials. The NALP3 inflammasome is a central activator of the innate immune defense in response to cellular infections and is capable of cleaving pro caspase-1 obtaining the biological active caspase-1. Caspase-1 in its active form cleaves the inactive pro Interleukin-1β (IL-1β), generating mature IL-1β [15]. It has been suggested, that NMs cause lysosomal rupture upon particle phagocytosis leading to ROS release into the cytoplasm and subsequent NALP3 inflammasome activation [16,17,18]. Therefore, IL-1β, as product of NALP3 inflammasome activation, can be seen as marker for particle induced inflammation.

In crystalline silica-exposed lungs, macrophages have emerged as key players in NALP3-mediated inflammation and tissue remodeling, although a contribution of structural epithelial cells cannot be excluded [19]. In the case of TiO_2_ NMs, the literature provides contrasting data regarding inflammasome activation in professional phagocytes of the innate immune system. Whereas some research groups detected an increased secretion of IL-1β from TiO_2_ treated bone marrow derived macrophages (BMDMs) [20,21], human THP-1 macrophage-like cells [8,22] or bone marrow derived dendritic cells (BMDCs) [23], other studies did not indicate an up-regulated release following TiO_2_ treatment in mouse RAW 264.7 cells [24], NR8383 rat alveolar macrophages [25] or BMDMs [26]. At least to some extent, these differences may be explained by differences in the cell-type used, but they could also relate to the selected type and even batch of the TiO_2_ nanomaterial as well as the method of their application to the cell system.

The aim of our study was, therefore, to determine to what extent TiO_2_ NMs are capable of activating the NALP3 inflammasome in macrophages. We selected a large panel of different TiO_2_ NMs and compared their effects against each other as well as against a sample of fine TiO_2_, a synthetic amorphous SiO_2_ and the well-investigated crystalline silica sample DQ12. The investigations were performed with BMDMs obtained from NALP3-deficient and NALP3 proficient mice as well in the well-established rat alveolar macrophages cell line NR8383. Our study findings are discussed in relation to current debate on the toxicity of NMs, specifically, regarding the inflammatory and inflammasome activating properties of TiO_2_ NMs in the lung.

## 2. Materials and Methods

### 2.1. Particles

For this study we selected a total of 19 different TiO_2_ NMs (abbreviated in this study as NT1 to NT19). The selected NMs have been the subject of investigation in various nanosafety and metrology projects. Our main experiments were performed with four TiO_2_ NMs, referred to as NT1 to NT4. Their origin and pristine characteristics are listed in Table 1. The samples NT1 to NT3 are three commercial samples, characterized by nearly spherical primary nanoparticles. Sample NT4 represents a truncated bipyramid shape TiO_2_ NM with an aspect ratio of 3:2, which has been synthesized by the University of Turin (Turin, Italy). Together with seven further test samples (i.e., NT5 to NT11) these NMs were all purchased or synthesized, and subsequently characterized, within the framework of the EU FP7 metrology project SetNanoMetro [27,28,29]. The other TiO_2_ NMs used in our study included the JRC repository samples NM100 (=NT12), NM101 (=NT13), NM103 (=NT14) and NM104 (=NT15) characterized and previously studied within the SIINN ERANET project NanOxiMet (for characteristics, see: https://www.nanopartikel.info/projekte/era-net-siinn/nanoximet/) and a set of TiO_2_ NMs that was characterized and investigated within the FP7 project ENPRA, i.e., NM101 (=N16), NRCWE001 (=NT17), NRCWE002 (=NT18) and NRCWE 003 (=NT19) (for characteristics, see: [30,31]). As negative control, a well-investigated fine TiO_2_ (=FT) was included [23,25]. Finally, amorphous silica (=AS) and fine crystalline silica (CS) were used as well-established positive controls [13,17,32]. The FT was obtained from Sigma Aldrich, Taufkirchen, Germany and represents a pure anatase sample with a BET surface area of 10 m^2^/g and a reported mean diameter of about 250 nm. The AS was purchased from Sigma Aldrich (#S5130), Taufkirchen, Germany, a fumed silica with a declared primary particle size of 7 nm and mean surface area of 395 ± 25 m²/g. The CS sample (DQ12 quartz, batch 6, IUF) has a mean particle size of 960 ± 620 nm, a surface area of 9.6 m²/g and a quartz content of 87% [33].

### 2.2. Isolation and Differentiation of Bone Marrow Derived Macrophages

The NALP3 inflammasome activating properties of the TiO_2_ NMs in macrophages were investigated using BMDM from mice. Therefore, C57BL/6J mice, originally purchased from Jackson Laboratories, Bar Harbor, ME, USA, were obtained from in-house breeding. In addition, B6.129S6-Nlrp3tm1Bhk/J mice were purchased from Jackson Laboratories [34]. For the preparation of BMDMs, mice with an age of 4 to 10 months were used. The mice were maintained according to the guidelines of the Society for Laboratory Animals Science (GV-SOLAS). The experiments (i.e., organ removal) were approved by the State Office for Nature, Environment and Consumer Protection of North Rhine-Westphalia, Germany (Landesamt für Natur, Umwelt und Verbraucherschutz, LANUV reference 84-02.05.40.14.138). The mice were sacrificed either by cervical dislocation or via i.p. injection of Phenobarbital (Narcoren^®^, Merial GmbH, Hallbergmoos, Germany, 800 mg/kg b.w.). Both hind legs were amputated, muscles as well as connective tissues were removed and the femur was cut and flushed with cold PBS from distal to proximal. Bone marrow of both femurs was combined and dispersed until cells were sufficiently separated. Afterwards, cells were passed through a 100 µm cell strainer to separate debris. The strainer was washed with PBS and cells were centrifuged at 800× *g* for 5 min. The cell pellet was re-suspended in 10 mL complete differentiation medium, consisting of RPMI 1640 Medium, containing 10% FCS, 10% L929 supernatant, 2% glutamine, 1% Penicillin/Streptomycin and 0.1% β-mercaptoethanol. Next, 5 × 10^6^ cells in 10 mL culture medium were seeded into a 100 mm bacteria culture dish and incubated at 37 °C and 5% CO_2_. After three days, 10 mL complete differentiation medium were added to each culture dish. At day six, adherent fraction represented the differentiated macrophages. To determine the success of the differentiation process, cells were stained with antibodies against F4/80 and CD11b and were analyzed by fluorescence activated cell sorting (FACS). Viability of all analyzed cells was between 77.2% and 95.8% and the percentage of differentiated macrophages reached from 77.5% to 97%. One day prior to each experiment, cells were seeded in a concentration of 5 × 10^4^ cells/cm^2^ in 96-well plates and incubated at 37 °C and 5% CO_2_.

### 2.3. NR8383 Cells

The NR8383 rat alveolar macrophage cell line, obtained from ATCC ((CRL-2192), Manassas, VA, USA) was cultured in DMEM/F-12 medium (Thermo Fisher Scientific, Waltham, MA, USA) containing 15% FCS, 1% penicillin/streptomycin and 1% glutamine (all purchased from Sigma-Aldrich, Taufkirchen, Germany) and incubated at 37 °C and 5% CO_2_. Two days prior to each experiment, cells were seeded at a density of 4 × 10^4^ cells/cm^2^ in 96-well plates and incubated at 37 °C and 5% CO_2_.

### 2.4. Treatment of Cells

The particle suspensions were prepared by dispersion in HLPC grade water at a concentration of 2 mg/mL and then sonicated with a Cuphorn (Branson Sonifier 450, Brookfield, CT, USA) for 10 min (Duty cycle 20%, power 5.71 (200 W)). Final particle concentrations were achieved by dilution of the particle suspensions with cell culture medium. The NR8383 cells were treated 48 h after seeding with particles at the indicated concentrations in medium without serum and phenol red whereas the BMDMs were treated in complete (i.e., FCS containing) medium 24 h after seeding. The experiments in the NR8383 cells were performed in the absence of FCS to abrogate proliferation of this immortalized cell line. As such, this in vitro model better reflects the typical non-proliferative phenotype of the resident macrophages of the lung alveoli. Moreover, it avoids a dose dilution over treatment time as would occur with proliferating cells in terms particle mass (or number) per unit cell number.

### 2.5. Dynamic Light Scattering

The dispersion states of the NMs in cell culture media were evaluated by dynamic light scattering (DLS) using a Delsa-Nano C (Beckman Coulter Inc., Krefeld, Germany). The measurements were performed for three types of suspensions relating to the specific dispersion protocol (including sonication and the respective culture media used for the NR8383 cells and BMDMs). Cumulative diameter and polydispersity index were determined for the four used TiO_2_ NMs (NT1-NT4) as well as the synthetic amorphous silica (AS), after suspension in dH_2_O or the DMEM and RPMI medium used for experiments. Results of these measurements are shown in Table 2, and represent mean values of three independent experiments.

### 2.6. WST-1 Assay

Cell viability was assessed using the WST-1 assay (Roche Diagnostics GmbH, Mannheim, Germany). Following 4 h or 24 h of particle treatment, two out of six replicates of each test condition were additionally treated with 1% Triton-X for 5 min, which served as positive control for maximal cell death and particle absorption. After addition of 10 µL WST-1 solution per well, cells were incubated for 2 h at 37 °C and 5% CO_2_. Afterwards, optical density was detected at 450 nm and 630 nm using a Thermo Multiskan GO Microplate Spectrophotometer (Thermo Fisher Scientific, Waltham, MA, USA) and percentage of mitochondrial activity related to the control was calculated. Particle absorption in cell-free samples was detected and subtracted from the calculated values of particle treated cells to exclude particle related effects.

### 2.7. IL-1β ELISA

For the assessment of inflammasome activation, 5 × 10^5^ cells per well of a 96 well plate were pre-treated with 10 (BMDMs) or 100 (NR8383) ng/mL lipopolysaccharide (LPS) for 4 h, to induce pro-IL1 β transcription. The respective priming concentrations of the LPS were selected on the basis of pilot experiments using CS (data not shown). Following LPS-priming, the cells were exposed to the different particles for 4 h or 24 h at the indicated concentrations. Cell free cell culture supernatants were collected and the amount of secreted IL-1β was detected by ELISA on a Thermo Multiskan GO Microplate Spectrophotometer (Thermo Fisher Scientific, Waltham, MA, USA), using commercial detection kits for mouse (i.e., BMDMs) (Bio-Techne Corporation, Minneapolis, MN, USA) or rat (i.e., NR8383 cells) (R&D Systems #RLB00). 

### 2.8. mRNA Expression Analyses by qRT-PCR

NR8383 cells were seeded in 6-well plates, treated with particles for 4 h, scraped and centrifuged (200 g, 5 min, 4 °C). The pellet was resuspended in 0.5 mL Trizol^®^ Reagent (Invitrogen GmbH, Karlsruhe, Germany) and stored at −80 °C until further use. For RNA extraction, 200 µL chloroform were added to each sample and incubated for 3 min at RT followed by centrifugation at 12,000 rcf at 4 °C for 15 min to separate the phases. The aqueous phase was transferred to a new tube and 400 µL Isopropanol were added. Samples were incubated for 10 min at room temperature followed by centrifugation at 12,000 rcf and 4 °C for 15 min. The RNA pellet was further washed using 75% ethanol and centrifugation for 5 min at 7500 rcf. RNA pellet was air dried and re-suspended in RNase-free water. Finally, samples were incubated for 10 min at 60 °C and purity of RNA was evaluated using spectrophotometry at 260 and 280 nm. cDNA was synthesized using the iScript™ cDNA Synthesis kit (BioRad, Hercules, CA, USA. cDNA was diluted 15× in RNAse-free water before use. Primer sequences for Heme oxygenase-1 (HO-1) were 5′-GGG AAG GCC TGG CTT TTTT -3′ (forward) and 5′-CAC GAT AGA GCT GTT TGA ACT TGGT -3′ (reverse), for inducible Nitric Oxide Synthase (iNOS) 5′- AGG AGA GAG ATC CGG TTC ACA GT -3′ (forward) and 5′- ACC TTC CGC ATT AGC ACA GAA -3′ (reverse), for IL-1β 5′-CAG GAA GGC AGT GTC ACT CA-3′ (forward) and 5′-AAA GAA GGT GCT TGG GTC CT -3′ (reverse), for IL-6 5′-GCC CTT CAG GAA CAG CTA TGA-3′ (forward) and 5′-TGT CAA CAA CAT CAG TCC CAA GA-3′ (reverse) and for β-actin 5′-CCC TGG CTC CTA GCA CCA T-3′ (forward) and 5′-ATA GAG CCA CCA ATC CAC ACA GA-3′ (reverse). qRT-PCR was performed with a MyiQ Single Color real time PCR detection system (BioRad, Hercules, CA, USA) using iQ™ SYBR^®^ Green Supermix (Biorad), 5 µL diluted cDNA, and 2.5 µL of 0.3 μM forward and reverse primer in a total volume of 20 µL. PCR was conducted as follows: a denaturation step at 95 °C for 3 min was followed by 40 cycles at 95 °C (15 s) and 60 °C (45 s). After PCR, a melt curve (55–95 °C) was generated for product identification and purity. Data were analyzed using the MyiQ Software system (BioRad) and were expressed as relative gene expression (fold increase) using the 2^−ΔΔCt^ method of [35] with β-actin as house-keeping gene.

### 2.9. ROS Measurement by Electron Paramagnetic Resonance (EPR) Spectroscopy

The amount of ROS formed in the treated BMDMs was evaluated using EPR spectroscopy with the use of the spin-trapping compound 5,5-dimethylpyrroline N-oxide (DMPO). Therefore, 31.25 × 10^5^ BMDMs per well of a 24 well plate were either primed for 4 h with LPS (10 ng/mL) or left un-primed. After 4 h, treatment medium was replaced by 100 µL Hank’s Balanced Salt Solution (HBSS) ^(+/+)^. Thereafter, 40 µg/cm^2^ of particles or 6 mM H_2_O_2_ (as positive control) were added, followed by the addition of 0.1 M DMPO (Sigma-Aldrich, Taufkirchen, Germany). After 1 h or 3 h incubation at 37 °C and 5% CO_2_, cell-free supernatants were harvested and immediately measured for radical formation using a MiniScope MS200 Spectrometer (Magnettech, Berlin, Germany) as described in [25]. Quantification was carried out on first derivation of EPR signal of the characteristic DMPO-OH quartet, as the mean of amplitudes, and outcomes are expressed in arbitrary units (a.u.).

### 2.10. Statistical Analyses

Statistical significances of experimental results were calculated by one-way analysis of variance (ANOVA) followed by Dunnett’s multiple comparison test for comparison of multiple treatments to the control. Student’s t-test was used for detection of significant differences between knock out and wild type macrophages. Significance was ascribed at *p* < 0.05. Analyses were conducted using SPSS statistics, Version 22 (IBM Corporation, Armonk, NY, USA).

## 3. Results

### 3.1. Effects of TiO_2_ NMs in BMDMs

**Figure 1:**
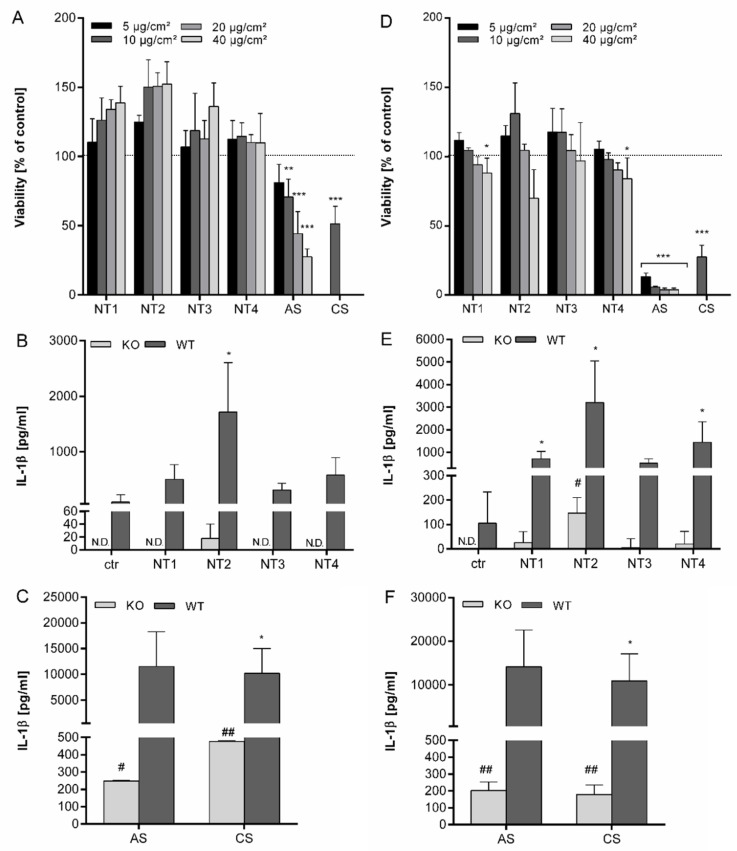
Effects of different TiO_2_ NMs on viability and IL-1β secretion after 4 h and 24 h in BMDMs. BMDMs were pre-incubated for 4 h with 10 ng/mL LPS and afterwards treated for 4 h or 24 h with 5–40 µg/cm² of four different TiO_2_ NMs (NT1, NT2, NT3, NT4) as well as amorphous SiO_2_ (AS) or crystalline silica (CS). Viability was detected using WST-1 assay and calculated as percent of control after treatment for 4 h (**A**) or 24 h (**D**). BMDMs of wild type and NALP3 knock out cells were treated for 4 h (**B**,**C**) or 24 h (**E**,**F**) with 40 µg/cm² of TiO_2_ NMs (**B**,**E**) or amorphous and crystalline silica (**C**,**F**). Release of IL-1β into culture supernatants was detected via ELISA. Mean and standard deviation of three independent experiments are depicted. The asterisks indicate a significant decrease in viability compared to untreated controls or significant increase in IL-1β concentrations compared to wild type controls. (* *p* ≤ 0.05; ** *p* ≤ 0.01; *** *p* ≤ 0.001). The hashes indicate a significant increase of IL-1β concentrations compared to untreated knock-out control cells (# *p* ≤ 0.05; ## *p* ≤ 0.01).

The ability of NMs to induce maturation and subsequent release of IL-1β, as detected by ELISA, is in support of their ability to activate the inflammasome pathway. Therefore, the BMDMs were pre-stimulated with LPS to induce transcription of the pro-form of IL-1β and subsequently treated with the various particles [23]. First, the effects of these treatment conditions on cellular viability were evaluated. BMDMs obtained from C57BL/6J mice were treated for 4 h with 10 ng/mL LPS and afterwards treated for 4 h and 24 h with 5 to 40 µg/cm^2^ of the particles (see Figure 1A,D). After 4 h treatment, only the viability of cells treated with the amorphous SiO_2_ (AS) or crystalline silica (CS) was significantly decreased. None of the four TiO_2_ NMs caused a decrease in cellular viability for this treatment time interval. After 24 h, however, viability was also significantly decreased by both NT1 and NT4 at the highest treatment concentrations (40 µg/cm^2^). The viability of the BMBMs was further decreased by AS and CS at 24 h compared to 4 h of treatment.

The release of IL-1β from the LPS pre-activated BMDMs by the different TiO_2_, AS and CS particles was then evaluated. Results are shown in Figure S1 (Supplementary Materials). Clear dose-dependent increases in IL-1β release were found for all investigated particles after 24 h treatment, suggestive of their ability to activate the NALP3 inflammasome in macrophages. However, large differences in IL-1β release were observed for the different particle types. The strongest responses were observed with AS and CS. The TiO_2_ particles showed much lower responses. Among them, NT2 revealed a markedly stronger effect than the other three TiO_2_ NMs. NT3 appeared the least active nanomaterial.

Importantly, even after adjustment of the differences in surface area, the differences in potency of the TiO_2_ NMs versus the amorphous SiO_2_ remained obvious. With a BET of 86 m^2^/g, the specific surface area of the NT2 is 9-fold higher than that of the CS (9.6 m^2^/g) and 5-fold lower than that of the AS (395 m^2^/g). Thus, when expressing the dose as BET surface area per cell culture dish area, the AS still caused an at least 2.5-fold higher IL-1β release than NT2. Similarly, this also demonstrated a much stronger IL-1β releasing response for CS compared to the amorphous AS.

To further explore if TiO_2_ NMs activate the inflammasome and to verify if they act differently in this activation, the release of IL-1β was then compared using BMBMs from NALP3 deficient and NALP3 proficient mice. Supernatants from BMDMs from both backgrounds were therefore analyzed in parallel after 4 h and 24 h treatment with all particles at equal mass dose (i.e., 40 µg/cm^2^). Results are shown in Figure 1. In the absence of LPS priming, no IL-1β release was detectable, neither in controls, nor in the particles treated BMDMs (data not shown). Using the LPS-priming protocol, IL-1β concentrations were increased for all four TiO_2_ NMs after 4 h, although this was significant only for NT2 (Figure 1B). The increase after treatment with NT3 was much lower than after treatment with NT2, confirming strong differences between the TiO_2_ NMs. Furthermore, 4 h treatment with AS and CS led to a much stronger increase of IL-1β secretion, which was about 5 times higher than for NT2 (Figure 1C). After 24 h, concentrations of IL-1β further increased and were significant for NT1, NT2 and NT4 (Figure 1E). Also, at this time, differences in IL-1β stimulating properties remained obvious among the four TiO_2_ samples, as well as in comparison to the AS and CS (Figure 1F).

Using NALP3 deficient cells IL-1β release after 4 h was only detectable for NT2, and at a higher level for AS and CS (Figure 1B and C). However, secretion was up to 20 times lower than in the BMDMs from wildtype mice, demonstrating the dependence of IL-1β secretion on the NALP3 inflammasome. After 24 h, IL-1β levels were detectable in the supernatants from NALP3 knockout cells after treatment with all particles. Effects were significant for NT2, AS and CS.

**Figure 2:**
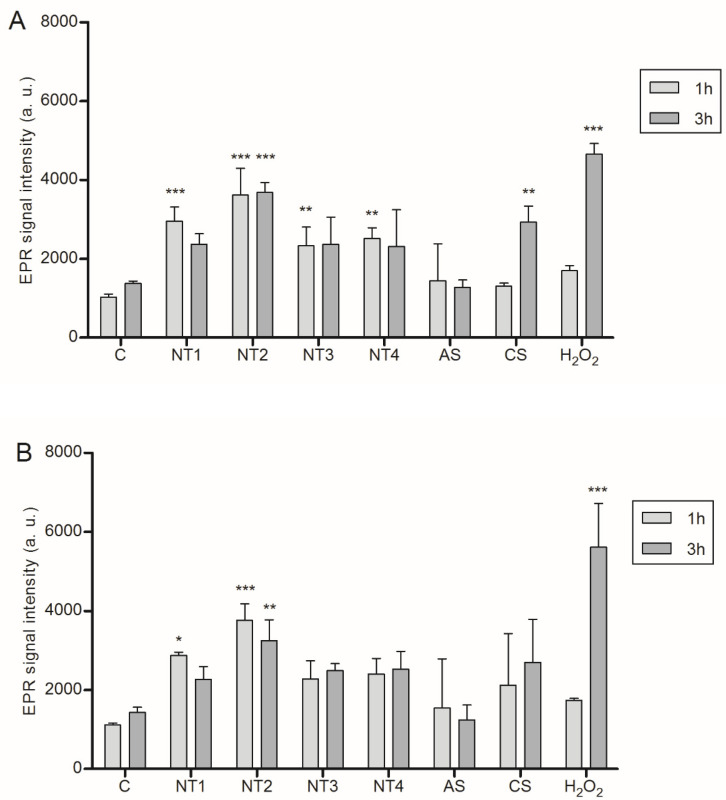
Detection of reactive oxygen species in BMDM via EPR spectroscopy. Cells were either primed for 4 h with 10 ng/mL LPS (**A**) or left unprimed (**B**) prior to treatment with 40 µg/cm² of the four TiO_2_ NMs, SiO_2_ or DQ12 for 1 or 3 h. As a positive control H_2_O_2_ (6 mM) was used. Simultaneously with these treatments, DMPO was added at a final concentration of 0.1 M. Mean and standard deviation of three independent experiments are depicted. The asterisks indicate a significant change in EPR signal intensity compared to untreated control (* *p* ≤ 0.05; ** *p* ≤ 0,01; *** *p* ≤ 0.001).

The role of ROS in inflammasome activation in the BMDMs was then explored by EPR spectroscopy. Results of this analysis are shown in Figure 2. Significant ROS increases were detected in LPS pre-activated BMDMs following treatment with all four TiO_2_ NMs as well as with CS. Interestingly, no significant increase was seen with AS. Without LPS pre-stimulation, significantly increased EPR signals were observed only with NT1 and NT2. Apart from the positive control H_2_O_2_, the strongest ROS response was always observed with NT2 (see Figure 2).

### 3.2. Effects of TiO_2_ NMs on NR8383 Rat Alveolar Macrophages

**Figure 3:**
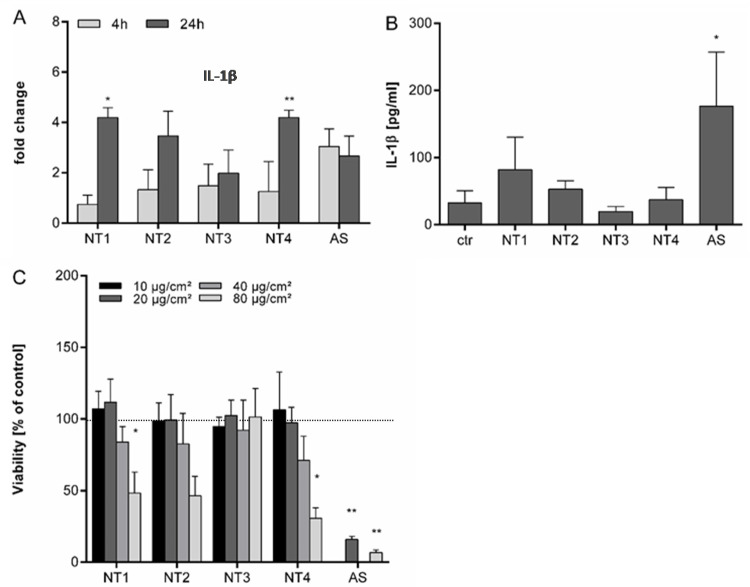
Effects of different TiO_2_ NMs on viability, IL-1β mRNA expression and release of IL-1β from NR8383 cells. NR8383 rat alveolar macrophages were treated for 4 h or 24 h with 40 µg/cm² of the four different TiO_2_ NMs (NT1-4) or amorphous silica (AS). Afterwards, fold changes in IL-1β mRNA expression were analyzed by qRT-PCR in relation to control cells (**A**). For detection of IL-1β in culture supernatants, cells were treated for 24 h and concentrations of IL-1β were detected by ELISA (**B**). Cell viability was evaluated by WST-1 assay following 24 h exposure to TiO_2_ NMs (NT1-4) at 10, 20, 40 or 80 µg/cm² or AS at 20 or 80 µg/cm² (**C**). Mean and standard deviation of three independent experiments are depicted. The asterisks indicate a significant difference in gene expression (**A**), IL-1β secretion (**B**) or viability (**C**) compared to untreated controls. (* *p* ≤ 0.05; ** *p* ≤ 0.01).

For the further evaluation of the inflammatory properties of the TiO_2_ NMs, experiments were performed with NR8383 rat alveolar macrophages. To evaluate potential direct effects of the NMs on IL-1β mRNA expression and protein release, experiments were performed in the absence of LPS priming. Results are shown in Figure 3. In anticipation of the higher robustness of these immortalized cells compared to the primary BMDMs, particle concentrations from 10 to 80 µg/cm² were selected for viability analysis by WST-1 assay. A significant reduction of viability was observed following 24 h treatment at 80 µg/cm^2^ for NT1 and NT4 (see Figure 3C). Although cytotoxicity levels are lower in the NR8383 cells, differences between the four TiO_2_ are comparable to the results observed with the BMDMs. Accordingly, we choose a treatment concentration of 40 µg/cm^2^ for further experiments with the NR8383 cells. As shown in Figure 3A, none of the TiO_2_ NMs caused a significant increase in IL-1β mRNA after 4 h of treatment. After 24 h treatment, mRNA levels were significantly increased for NT1 and NT4. Increases in mRNA expression were also noted for NT2 at 24 h, and for AS at both 4 h and 24 h, although these effects did not reach statistical significance. Effects of the particles on IL-1β release from the NR8383 cells are shown in Figure 3B. In the absence of LPS priming, a significant increase in IL-1β release was only observed for AS. IL-1β levels were not significantly increased by the TiO_2_ NMs. Subsequent analysis of tumor necrosis factor (TNF) levels in the supernatants revealed findings that were in concordance with the IL-1β protein data. Results are shown in Figure S2 (Supplementary Materials). A significant increase of TNF was observed for AS, but not for TiO_2_ NMs.

**Figure 4:**
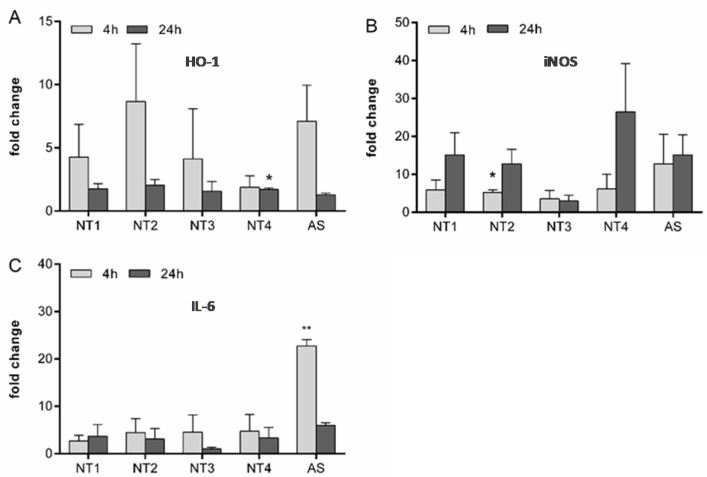
Effects of different TiO_2_ NMs on mRNA expression of HO-1, iNOS and IL-6 in NR8383 cells. For analysis of changes in the mRNA regulation of HO-1 (**A**), iNOS (**B**) and IL-6 (**C**), NR8383 cells were treated for 4 h or 24 h with 40 µg/cm² of the four TiO_2_ NMs (NT1-4) or amorphous silica (AS). Afterwards, mRNA levels were detected via qRT-PCR. Mean and standard deviation of three independent experiments are depicted. The asterisks indicate a significant difference in gene expression compared to untreated controls. (* *p* ≤ 0.05; ** *p* ≤ 0.01).

To further explore the observed variability in the inflammatory properties of the TiO_2_ NMs, the mRNA expression of HO-1, iNOS and IL-6 were evaluated (Figure 4). For the oxidative stress marker gene HO-1, the most pronounced upregulations were observed with NT2 and amorphous silica after 4 h treatment. At this time point, there were substantial differences in HO-1 mRNA expression between the four TiO_2_ NMs (see Figure 4A). After 24 h, the mRNA levels of HO-1 tended to decline to control levels for all used particles. In contrast, the mRNA expression of iNOS increased with increased treatment time. At 24 h, at least 10-fold increased levels were found for all NMs except NT3 (see Figure 4B). Analysis of the mRNA expression of the inflammatory cytokine IL-6 revealed a marked upregulation at 4 h for AS. This effect seemed to be transient as indicated from the lower mRNA levels observed at 24 h for this sample. No such marked effects on IL-6 mRNA expression were observed for the TiO_2_ NMs.

### 3.3. Evaluation of the Inflammasome Activating Capacity of a Panel of 19 TiO_2_ NMs

**Figure 5:**
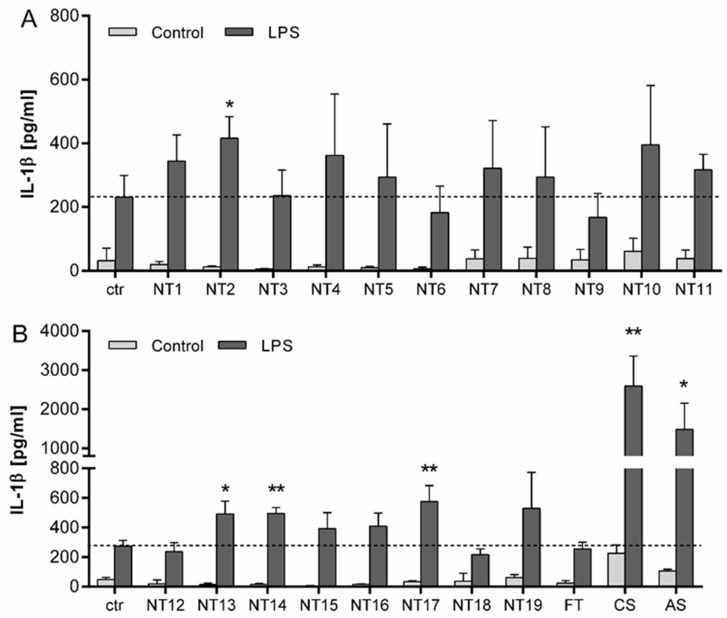
IL-1β release from NR8383 cells following 24 h treatment with TiO_2_ NMs. Cells were either primed for 4 h with 100 ng/mL LPS or left without priming, followed by 24 h treatment with 40 µg/cm² of different particles. (**A**) IL-1β levels in NR8383 cells treated with TiO_2_ NMs (NT1–NT11) from the SetNanoMetro project. (**B**) IL-1β levels in NR8383 cells treated with the TiO_2_ NMs from the projects NanOxiMet (NT12- NT15), ENPRA (N16-N19), fine TiO_2_ (FT), amorphous SiO_2_ (AS) and the crystalline silica sample DQ12 (CS). IL-1β concentrations were analyzed by ELISA. Mean and standard deviation of three independent experiments are depicted. The dashed lines represent the IL-1β concentrations released from control cells. For statistical analysis, the mean values of unprimed cells were subtracted from LPS primed mean values. The asterisks indicate a significant change in IL-1β concentration compared to untreated control (* *p* ≤ 0.05; ** *p* ≤ 0.01).

To determine the effects of TiO_2_ with different characteristics on the secretion of IL-1β, LPS primed NR8383 cells were treated with 40 µg/cm² of a large panel of different TiO_2_ NMs for 24 h (Figure 5). Compared to control cells, IL-1β concentrations in particle treated cells without priming were not significantly increased. Cells pre-treated with 100 ng/mL LPS without subsequent nanoparticle treatment secreted an increased amount of IL-1β. The additional treatment with the different TiO_2_ NMs led to contrasting results. While treatment with some of the used NMs (i.e., NT2, NT13, NT14 and NT17) led to significantly increased IL-1β concentrations in the cell culture supernatant, others did not show comparable effects. In fact, for five out of the nineteen TiO_2_ NMs, IL-1β concentrations in the supernatant of cells were not elevated at all (i.e., NT3, NT6, NT9, NT12, NT18). Also, the fine TiO_2_ particles that were included in these experiments failed to induce any IL-1β release. In contrast, AS as well as CS showed highly significant increases in IL-1β secretion into the supernatant of the LPS-primed NR8383 cells. Taken together, these data confirmed a considerable diversity of IL-1β secretion for the different TiO_2_ NMs used in this study.

## 4. Discussion

There has been substantial controversy and debate on the toxic and pro-inflammatory properties of TiO_2_ NMs, specifically regarding their ability to activate the NALP3 inflammasome. Here, we evaluated the inflammasome-activating properties of a panel of 19 different TiO_2_ NMs alongside with a fine crystalline silica and a synthetic amorphous silica sample. The release of IL-1β, as product of NALP3 inflammasome activation was analyzed from mouse BMDMs as well as NR8383 rat alveolar macrophages with or without LPS pre-activation. The BMDMs were included in this study for two reasons. First, the available literature indicates that primary phagocytes, like BMDMs or BMDCs, are more sensitive to TiO_2_ NMs than immortalized cell lines regarding IL-1β release [20,21,23]. Moreover, the use of BMDMs allowed us to directly compare the IL-1β releasing properties of the NMs in primary cells from mice with or without functional NALP3 inflammasome.

In the NALP3 proficient BMDMs, increased IL-1β release was observed for TiO_2_ NMs upon LPS pre-treatment, albeit at different levels among the four investigated samples. First, our findings demonstrate that nanoparticulate TiO_2_ is able to induce the release of IL-1β from macrophages at sub-cytotoxic concentrations, as verified by WST-1 assay. Second, the contrasting levels of IL-1β release suggest that the ability of TiO_2_ NMs to trigger NALP3 inflammasome activation depends on their particle characteristics. Comparable results were observed by other investigators [8], using the human macrophage-like cell line THP-1. In our hands, effects of the amorphous silica and the crystalline silica on IL-1β release were much stronger than the effects of the TiO_2_ NMs at equal mass dose. This is in support of the strong difference in the inflammatory potency between these respective particle types. Considering the surface area as dose metric, the IL-1β releasing potency of the crystalline silica is markedly higher than that of the amorphous silica while that of the TiO_2_ NMs was lower than that of the amorphous SiO_2_. A strong difference in surface area-adjusted inflammatory potency between crystalline silica and TiO_2_ NMs was previously also found for interleukin-8 release from lung epithelial cells [10]. For the four TiO_2_ NMs, the IL-1β secretion after 24 h treatment was higher compared to 4 h treatment, revealing a time dependent effect of this pro-inflammatory response. However, levels of IL-1β at 24 h were only twice as high as after 4 h treatment. Also for amorphous and crystalline silica the levels of IL-1β did not differ substantially between 4 h and 24 h. These findings are in alignment with the role of IL-1β as an early mediator in inflammation [36].

The NALP3 inflammasome dependence of the IL-1β secretion by the TiO_2_ NMs was demonstrated by our findings with the BMDMs of NALP3 deficient mice. Compared to the BMDMs from the NALP3 proficient mice a massive reduction of IL-1β release was observed with the deficient macrophages. Our findings also point towards an alternative, NALP3 independent mechanism of IL-1β processing, as indicated by the time-dependent IL-1β release from knockout BMDMs upon treatment with NT2 (as well with the amorphous and crystalline silica). This mechanism possibly involves a direct processing of pro IL-1β by cathepsins [37]. The experiments with NALP3 deficient macrophages also confirmed the well-established inflammasome activating capacity of crystalline silica [13,17,19] as well of amorphous silica [38,39,40]. Complementary investigations with a commercial luminescence-based casapase-1 activity kit provided further support for the contrasting inflammasome activating properties of these different particle types. In supernatants of NALP3-proficient BMDMs we found no significant increases in caspase-1 activity upon treatment with the TiO_2_ NMs in contrast to the crystalline silica, while an intermediate effect was observed for the amorphous silica sample (data not shown). Unfortunately, we could not reliably analyze caspase-1 activity within BMDM cell lysates, likely as a results of assay interference with endocytosed particles. Further research is needed to elaborate on the mechanisms of NALP (in)dependent IL-1β maturation in particle exposed BMDMs.

Generation of ROS has been linked to the inflammatory properties of NMs, including their ability to activate the NALP3 inflammasome [16,17,18,40]. Therefore, we also determined ROS levels in the particle treated BMDMs by EPR. Indeed, increased ROS levels were observed following treatment with the TiO_2_ NMs. Moreover, the strongest effect was observed for NT2, the sample that also showed the strongest NALP3-dependent IL-1β release, in support of the role of ROS in NALP3 inflammation by nanoparticulate TiO_2_. The involvement of ROS was further substantiated by complementary investigations, revealing a significant inhibition of IL-1β release in NT2 treated BMDMs that were pre-treated with the antioxidant diphenyleneiodonium (DPI) (data not shown).

Notably, in the EPR analyses the ROS increases observed with the TiO_2_ NMs did not substantially differ from those observed with AS and CS, even though the latter two particles had a much more pronounced IL-1β response. This indicates that TiO_2_ NMs, AS and CS, at least in part, elicit NALP inflammation through distinct mechanisms. The marked differences in cytotoxicity between these types of particles and their impact on endogenous ROS sources should be taken into account here.

Our subsequent investigations were performed with NR8383 cells, a well-established alveolar macrophage cell line from rat [41]. NR8383 cells have emerged as a reliable in vitro model to study the mechanisms of toxicity of various particles including crystalline and amorphous silica as well as TiO_2_ particles [25,42,43]. Obviously, in vitro systems never fully recapitulate the in vivo situation but nevertheless can be very useful for hazard ranking. Indeed, the NR8383 cell line has also proven to be useful for (nano)particle hazard grouping strategies, showing good comparability with outcomes from in vivo studies [44,45,46]. To evaluate the suitability of the NR8383 cells for the investigation of the inflammasome activating properties of the TiO_2_ NMs, at first, experiments were performed in the absence of LPS priming. In this case, no increased IL-1β concentrations were found following treatment with any of the four TiO_2_ NMs. In contrast, treatment with amorphous SiO_2_ led to a significant increase in IL-1β secretion from the non-LPS-primed NR8383 cells. In concordance with this, also no significant release of the early inflammatory cytokine TNF was found with the TiO_2_ NMs, again in contrast to the amorphous SiO_2_ sample (Figure S2). Crystalline silica was not included in these NR8383 experiments, but its ability to induce IL-1β release (as well as TNF) in the absence of LPS-priming has been well-documented for this cell line [25,47]. Taken together, our data suggest that TiO_2_ NMs are incapable of inducing a full immunological activation without LPS priming. Activation of the IL1B gene by particles is mediated by the activation of the transcription factors nuclear factor-κB (NFκB) and activator protein 1 (AP-1) [17]. Accordingly, the mRNA expression analysis of the non-LPS-primed NR8383 cells further supported the poor activating properties of the TiO_2_ NMs. At early time point (4 h), elevated IL-1β mRNA levels were only observed with the amorphous SiO_2_. At 24 h, statistically significant increases in IL-1β mRNA expression were observed with NT1 and NT4 but overall the effects in the NR8383 cells were modest, in alignment with the poor ability of TiO_2_ to activate NFκB in these cells, shown previously [25].

To further analyze the effects of TiO_2_ NMs on oxidative stress induction and inflammation in the NR83838 cells, the mRNA expression levels of HO-1, iNOS and IL-6 were analyzed. HO-1 is considered as a sensitive marker of oxidative stress, and its mRNA expression has been found upregulated in lungs of crystalline-silica exposed rats [47] as well as mice [48]. In our present study, HO-1 mRNA levels were enhanced following 4 h treatment with the amorphous SiO_2_ and all TiO_2_ NMs, except NT4, indicative of oxidative stress by these NMs. The absence of marked increases in HO-1 mRNA expression levels at the 24 h time point likely relates to a compensatory upregulation of antioxidant defense responses [49,50]. In contrast, the mRNA levels of iNOS were most strongly increased after 24 h treatment for all NMs, with the exception of NT3. Considering the transcriptional activation of this gene as a marker of inflammation, as revealed for crystalline silica [47,51], current iNOS mRNA data fit well with the observed release of IL-1β. Both in the NR8383 cells and the BMDMs, NT3 caused the lowest IL-1β secretion among the four TiO_2_ NMs. Our current data also align with previous findings in our lab, showing increased mRNA expression of HO-1 as well as iNOS in NR8383 cells treated with crystalline silica and TiO_2_ NMs, while fine TiO_2_ particles were not reactive [25]. In our current study, unlike HO-1 and iNOS, the mRNA expression analysis of IL-6 did not reveal marked contrasts between the different TiO_2_ NP. However, a substantial up-regulation was observed in the NR8383 cells treated with the amorphous silica. As such, these data align well with the secretion pattern of IL-1β (and TNFα), and further supports the contrasting pro-inflammatory potency of this nano-SiO_2_, when compared to the nano-TiO_2_.

The importance of the physicochemical properties of particles on inflammasome activation has been reviewed recently [17] and shown for TiO_2_ in THP-1 macrophages [8]. In our hands, neither the characteristics of the pristine samples (Table 1), nor their morphological behavior in suspensions (Table 2), provided any plausible explanation for the observed differences in the IL-1β generating potency of the TiO_2_ NMs. The two samples that showed the highest contrast in IL-1β release, i.e., NT2 and NT3, both were pure anatase phase and were also nearly identical in primary particle diameter as well as their specific particle surface area (BET). Furthermore, there was no apparent correlation between IL-1β release and the agglomeration states of the 4 samples. Here the strong differences in agglomeration state for the different culture media used for the BMDMs and the NR8383 should also be highlighted. As a result of the differences in media composition, the four TiO_2_ samples ranked differently regarding agglomerate size. Yet, for both cell types, NT2 turned out to be the most potent in terms of inflammasome activation and IL-1β release. The differences in agglomeration status of the TiO_2_ NMs in the different culture media can be explained by the absence of the FCS in the “DMEM” used for the NR8383 cells, and aligns with previous investigations by Allouni and coworkers [52].

This observation is all the more interesting, as it suggests that some TiO_2_ NMs can trigger IL-1β release from LPS primed macrophages, irrespective of the presence of an abundant amount of proteins in the cell culture medium. It has been well-established that the in vitro effects of NMs, including TiO_2_ and SiO_2_ may strongly depend on the presence of FCS [31,53,54,55]. Therefore, further research is warranted to determine the role of corona-forming constituents in the inflammasome pathway activating properties of inhaled TiO_2_ NMs, or other types of particles like the amorphous and crystalline silica that were used as positive control in our study. In this regard it will be particularly relevant to evaluate the effects of realistic lung lining fluids, such as the artificial bronchoalveolar lavage fluid (BALF) introduced by Porter and coworkers [56].

An independent property that possibly drives inflammasome activating properties of TiO_2_ is shape. Indeed, in a side-by-side toxicological evaluation of long versus shortened TiO_2_ nanofibers, Bianchi and colleagues recently showed an increased pro-inflammatory response in mouse lung and peritoneum for the longer samples [57]. Earlier, Porter et al. [58] evaluated the role of TiO_2_ NM shape and length upon pharyngeal application in mouse lung by comparison of the effects of TiO_2_ nanospheres and short and long nanobelts with respective aspect ratios of 1:30 and 1:80. They identified persistent inflammatory effects for the nanobelts, with the longer one being more potent and also exclusively causing significant lung tissue remodeling (i.e., fibrosis). While these studies demonstrated the importance of length in the pro-inflammatory potency of so-called high aspect ratio nanomaterials (HARN) composed of TiO_2_, they cannot provide a direct explanation for our current study findings. The TiO_2_ NMs samples in our study were composed of primary particles with all three external dimensions in the nanoscale and do not categorize as HARN (data not shown).

In contrast to the aforementioned physicochemical properties of the TiO_2_ NMs, their ability to induce ROS generation and associated oxidative stress was found to be a good predictor of the inflammasome activating properties in our hands. Both in the BMDMs and the NR8383 cells, the strongest IL-1β release in LPS-primed conditions was observed with the sample NT2. This sample also displayed the highest and most sustained ROS levels during treatment of the BMDMs, as well as the highest mRNA expression levels of the oxidative stress marker gene HO-1 in the NR8383 cells.

Finally, to prove the feasibility of NR8383 cells for the hazard screening of NMs, specifically regarding their inflammasome activating capacity, we selected a large panel of particles. In addition to the four in-depth-investigated TiO_2_ NMs, the amorphous silica and the crystalline silica sample DQ12, 15 further TiO_2_ NMs were included as well as a sample of fine TiO_2_. For the six samples that were studied in both in vitro models a remarkable concordance was found in terms of qualitative responses: The TiO_2_ sample NT3 did not cause an increased IL-1β release upon LPS priming in both cell models whereas NT2, and more importantly, the positive controls CS and AS did. Again, one should keep in mind here that the NR8383 cells were treated in the absence of FCS to inhibit undesired proliferation of this robust cell line, whereas the primary mouse BMDMs required treatment in FCS containing medium. Thus, while the effects appeared qualitatively similar, the contrasting abundance of corona forming proteins in the respective treatment media may very well explain for some of the observed quantitative differences in IL1β responses between both in vitro models. Moreover, differences in the constitutive or LPS-induced release of IL1β between cell lines and primary cells or between murine and human cells may also play a role.

The outcome of the NR8383 experiments with the large panel of particles further confirmed our observations about the variation in responsiveness of macrophages to TiO_2_ NMs. Indeed, the available literature already points to a heterogeneity in effects of TiO_2_ NMs. However, for our present findings we can rule out any potential effects that could result from differences in the selected macrophage cell type and its culturing protocol as well as the particle handling procedure and administration protocol. Among the 19 tested nano-TiO_2_ samples, four samples showed a significant increase in IL-1β release from the LPS-primed NR8383 macrophages while five nano-TiO_2_ samples did not show any increase above control. The remaining ten TiO_2_ NMs could be arbitrarily grouped as “intermediate”. Again, it should be emphasized that, while displaying a clear gradient in IL-1β releasing potency, the responsiveness of the NR8383 cells to amorphous and crystalline silica was much greater. Moreover, when considered per unit surface area, the fine crystalline silica was much more potent than the synthetic amorphous silica. Taken together, these data suggest that evaluation of the inflammasome generating properties in the immortalized NR8383 rat alveolar macrophage cell line is a useful approach for a pro-inflammatory hazard ranking of inhaled nanomaterials. This obviously includes the assessment of NMs of different classes and chemical composition, but likely also even within an individual group of NMs of the same chemical composition, as exemplified here by the case of TiO_2_.

## 5. Conclusions

Our study demonstrates that specific TiO_2_ nanomaterials can activate the inflammasome in macrophages in association with ROS generation, albeit with a markedly lower potency than the synthetic amorphous silica and the crystalline silica. The heterogeneity in IL-1β release among 19 different TiO_2_ samples observed in our study underscores the need for case-by-case evaluation of the inflammasome activating capacity of NMs. Our data also suggests that the NR8383 cell line can serve as a robust in vitro tool for such evaluation.

## Figures and Tables

**Table 1:** Characteristics of the pristine TiO_2_ nanomaterials.

Sample	ID	Supplier	Diameter ^1^ (nm)	BET ^2^ (m^2^/g)	Crystal Phase ^3^	Method of Synthesis
NT1	P25	Evonik	12–18	55	A/R	flame pyrolysis of TiCl_4_
NT2	PC105	Cristal	10	86	A	hydrolysis of titanyl sulfate and unspecified thermal treatment
NT3	SX001	Solaronix	12–15	93	A	hydrothermal process
NT4	UT001	UNITO ^4^	16–17	47	A	hydrolysis of aqueous solution of Ti^IV^(triethanolamine)_2_titanatrane

^1^ Primary particle size; ^2^ Specific surface area according to Brunauer Emmett and Teller; ^3^ A = Anatase/R = Rutile; ^4^ University of Turin.

**Table 2:** Nanoparticle characteristics by Dynamic Light Scattering.

	Cumulant Diameter ^1^			Polydispersity Index ^1^		
Sample	dH_2_O	DMEM	RPMI	dH_2_O	DMEM	RPMI
NT1	193 ± 11	2338 ± 55	250 ± 5	0.208 ± 0.03	0.467 ± 0.02	0.191 ± 0.01
NT2	650 ± 19	1188 ± 28	775 ± 17	0.222 ± 0.07	0.380 ± 0.004	0.275 ± 0.01
NT3	516 ± 118	1684 ± 73	476 ± 118	0.235 ± 0.05	0.530 ± 0.03	0.215 ± 0.05
NT4	207 ± 18	1987 ± 50	335 ± 44	0.135 ± 0.03	0.456 ± 0.02	0.154 ± 0.02
AS	181 ± 1.1	192 ± 2.7	338 ± 4.3	0.144 ± 0.02	0.174 ± 0.01	0.257 ± 0.02

^1^ Data represent mean ± SD (*n* = 3).

## Supplementary Materials

The following are available online at https://www.mdpi.com/2079-4991/10/9/1876/s1, Figure S1: IL-1β concentration in cell culture supernatants of BMDMs after 24 h treatment with different TiO_2_ NMs, amorphous silica and crystalline silica. Figure S2: Tumor Necrosis Factor concentrations in supernatants of NR8383 cell cultures after 24 h treatment with different TiO_2_ NMs and amorphous silica.
