# Supplementary material for: Evaluation of the NLRP3 Inflammasome Activating Effects of a Large Panel of TiO2 Nanomaterials in Macrophages
Source: Nanomaterials (Basel). 2020 Sep 19;10(9):1876. doi: 10.3390/nano10091876 (PMC7558067; doi:10.3390/nano10091876)
Supplement: Supplementary file 1 [file nanomaterials-10-01876-s001.pdf]

nanomaterials

SUPPLEMENTARY MATERIALS

Evaluation of the NLRP3 Inflammasome Activating Effects of a Large Panel of TiO_2 Nanomaterials in Macrophages

Julia Kolling [1], Jonas Tigges [1,2], Bryan Hellack [3,4], Catrin Albrecht [1,5] and Roel P. F. Schins [1,*]

1 IUF—Leibniz Research Institute for Environmental Medicine, Auf'm Hennekamp 50, 40225 DE Düsseldorf, Germany; julia.kolling@gmx.de (J.K.); jonas.tigges@gmx.de (J.T.); catrin.albrecht@sachsen-anhalt.de (C.A.)
2 Bundeswehr Institute of Pharmacology and Toxicology, Neuherbergstraße 11, 80937 DE Munich, Germany
3 Institute of Energy and Environmental Technology e.V. (IUTA), Bliersheimer Str. 60, 47229 Duisburg, Germany; bryan.hellack@uba.de
4 UBA—German Environment Agency, Paul-Ehrlich-Str. 29, 63225 Langen, Germany
5 State Office for Consumer Protection Saxony-Anhalt, 39576 Stendal, Germany
* Correspondence: roel.schins@uni-duesseldorf.de; Tel.: +49-211-3389-269

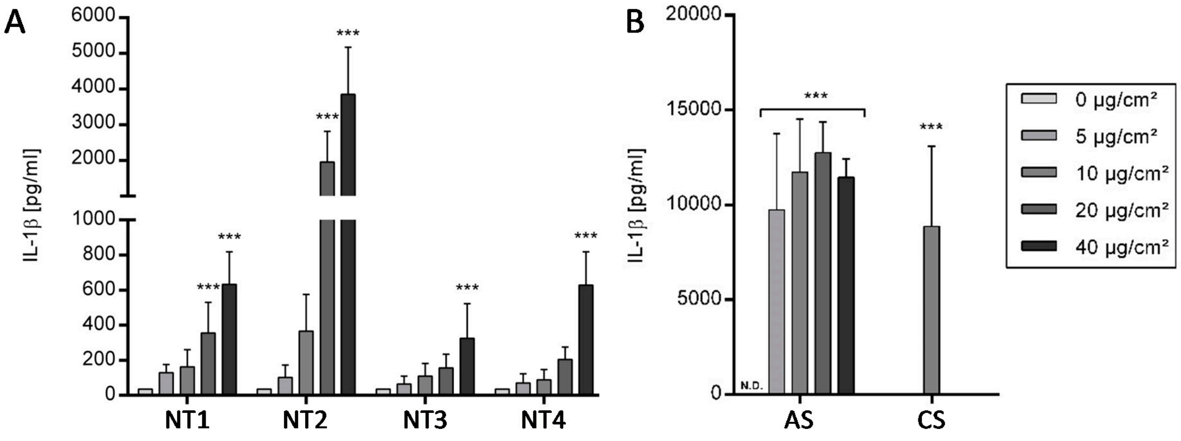

Figure S1. Interleukin-1β concentrations in cell culture supernatants of BMDMs after 24 h treatment with different TiO_2 NMs, amorphous silica and crystalline silica. BMDMs were primed for 4 h with 10 ng/ml LPS followed by 24 h treatment with 5–40 µg/cm² TiO_2 NMs (NT1, NT2, NT3, NT4) or amorphous SiO_2 (AS). The crystalline silica sample DQ12 (CS) was used as a positive control at a single concentration of 10 µg/cm². IL-1β concentrations in culture supernatants were analysed by ELISA. Mean and standard deviation of 3 independent experiments are depicted. The asterisks indicate a significant change in IL-1β concentration compared to untreated control (* $p \leq 0.05$; *** $p \leq 0.001$; N.D. = not detectable).

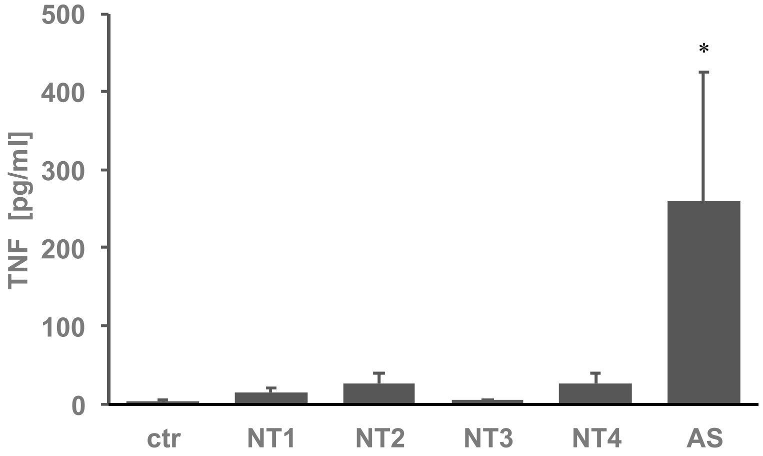

Figure S2. Tumor Necrosis Factor concentrations in supernatants of NR8383 cell cultures after 24 h treatment with different TiO_2 NMs and amorphous silica. The cells were treated for 24 h with 40 µg/cm^2 of the four different TiO_2 NMs (NT1-4) or amorphous silica (AS). Levels of TNF in culture supernatants were detected by a commercial rat ELISA kit according to the manufacturer's instructions (R&D/Biotechne, Minneapolis, MN, USA). Mean and standard deviation of three independent experiments are depicted. The asterisks indicate a significant difference compared to the untreated controls. (* $p \leq 0.05$).
